# Inferring gene regulatory networks from single-cell multiome data using atlas-scale external data

**DOI:** 10.1038/s41587-024-02182-7

**Published:** 2024-04-12

**Authors:** Qiuyue Yuan, Zhana Duren

**Affiliations:** Center for Human Genetics, Department of Genetics and Biochemistry, Clemson University, Greenwood, SC USA

**Keywords:** Gene regulatory networks, Dynamic networks

## Abstract

Existing methods for gene regulatory network (GRN) inference rely on gene expression data alone or on lower resolution bulk data. Despite the recent integration of chromatin accessibility and RNA sequencing data, learning complex mechanisms from limited independent data points still presents a daunting challenge. Here we present LINGER (Lifelong neural network for gene regulation), a machine-learning method to infer GRNs from single-cell paired gene expression and chromatin accessibility data. LINGER incorporates atlas-scale external bulk data across diverse cellular contexts and prior knowledge of transcription factor motifs as a manifold regularization. LINGER achieves a fourfold to sevenfold relative increase in accuracy over existing methods and reveals a complex regulatory landscape of genome-wide association studies, enabling enhanced interpretation of disease-associated variants and genes. Following the GRN inference from reference single-cell multiome data, LINGER enables the estimation of transcription factor activity solely from bulk or single-cell gene expression data, leveraging the abundance of available gene expression data to identify driver regulators from case-control studies.

## Abstract (web summary)

Accuracy of gene regulatory network inference is increased by combining multiome single-cell and atlas-scale bulk data.

## Main

GRNs^1,2^ are collections of molecular regulators that interact with each other and determine gene activation and silencing in specific cellular contexts. A comprehensive understanding of gene regulation is fundamental to explain how cells perform diverse functions, how cells alter gene expression in response to different environments and how noncoding genetic variants cause disease. GRNs are composed of transcription factors (TFs) that bind DNA regulatory elements to activate or repress the expression of target genes.

Inference of GRNs is a central problem^2–4^, and there have been many attempts to approach this issue^2,5–13^. Co-expression-based methods such as WGCNA^14^, ARACNe^9^ and GENIE3 (ref. ^15^) infer the TF–TG *trans*-regulation from gene expression by capturing the TF–TG covariation. Such networks have undirected edges, preventing distinction of direction from a TF_A_–TF_B_ edge. Moreover, co-expressions are interpreted as correlations rather than causal regulations^16^. Genome-wide measurements of chromatin accessibility, such as DNase-seq^17^ and assay for transposase-accessible chromatin sequencing (ATAC-seq)^18^, locate REs, enabling TF–RE connections by motif matching and connecting REs to their nearby TGs^19^. However, TF footprint approaches cannot distinguish within-family TFs sharing motifs. To overcome this limitation, we developed a statistical model, PECA^20^, to fit TG expression by TF expression and RE accessibility across a diverse panel of cell types. However, the problem still has not been fully resolved because heterogeneity of cell types in bulk data limits the accuracy of inference.

The advent of single-cell sequencing technology has enabled highly accurate regulatory analysis at the level of individual cell types. Single-cell RNA sequencing (scRNA-seq) data enables cell type-specific *trans*-regulation inference through co-expression analysis such as PIDC and SCENIC^21–30^. Single-cell sequencing assay for transposase-accessible chromatin (scATAC-seq) can be used to infer *trans*-regulation, as in DeepTFni^31^. Many methods integrate unpaired scRNA-seq and scATAC-seq data to infer *trans*-regulation. Those methods, including IReNA^32^, SOMatic^33^, UnpairReg^34^, CoupledNMF^35,36^, DC3 (ref. ^36^) and others^37^ link TFs to REs by motif matching and link REs to TGs using the covariation of RE–TG or physical base pair distance. Recently, scJoint^38^ was developed to transfer labels from scRNA-seq to scATAC-seq data, which may enable improved cell GRN inference. Despite extensive efforts, GRN inference accuracy has remained disappointingly low, marginally exceeding random predictions^39^.

Recent advances in single-cell sequencing^40^ provide opportunities to address these challenges^41^, exemplified by SCENIC+^42^. However, three major challenges persist in GRN inference. First, learning such a complex mechanism from limited data points remains a challenge. Although single-cell data offers a large number of cells, most of them are not independent. Second, incorporating prior knowledge such as motif matching into non-linear models is challenging. Third, inferred GRN accuracy assessed by experimental data is only marginally better than random prediction^39^.

To overcome these challenges, we propose a method called LINGER (Lifelong neural network for gene regulation). This research paper contributes to the field of GRN inference in multiple ways. First, LINGER uses lifelong learning, a previously defined concept^43^ that incorporates large-scale external bulk data, mitigating the challenge of limited data but extensive parameters. Second, LINGER integrates TF–RE motif matching knowledge through manifold regularization, enabling prior knowledge incorporation into the model. Third, the accuracy of LINGER represents a fourfold to sevenfold relative increase. Fourth, LINGER enables the estimation of TF activity solely from gene expression data, identifying driver regulators.

## Results

### LINGER: using bulk data to infer GRNs from single-cell multiome data

**Fig. 1:**
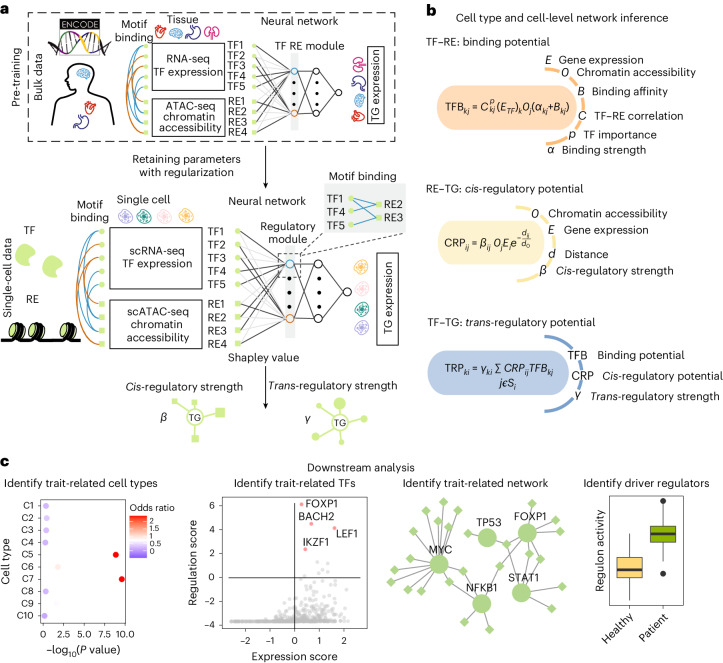
Schematic overview of LINGER. **a**, Schematic illustration of LINGER: a model predicting gene expression by TF expression and chromatin accessibility using a neural network model. LINGER pre-trains on the atlas-scale external bulk data and retains parameters by lifelong learning. The population-level GRN is generated from the neural network using the Shapley value. **b**, Strategy for constructing cell type-specific and cell-level GRNs. Cell type-specific and cell-level GRNs are inferred by an identical strategy, which combines consistent information across all cells, including regulatory strength, motif binding affinity and RE–TG distance, with context-specific information on gene expression and chromatin accessibility. **c**, Downstream analyses enabled by LINGER-inferred GRNs, including identifying complex regulatory landscape of GWAS traits and driver regulator identification.

LINGER is a computational framework designed to infer GRNs from single-cell multiome data (Fig. 1 and Methods). Using count matrices of gene expression and chromatin accessibility along with cell type annotation as input, it provides a cell population GRN, cell type-specific GRNs and cell-level GRNs. Each GRN contains three types of interactions, namely, *trans*-regulation (TF–TG), *cis*-regulation (RE–TG) and TF-binding (TF–RE). Note that TF–TF interactions are included in TF–TG pairs but TF self-regulation, which is challenging to model without additional data, is not considered. LINGER is distinguished by its ability to integrate the comprehensive gene regulatory profile from external bulk data. This is achieved through lifelong machine learning, also called continuous learning. The concept of lifelong learning is that the knowledge learned in the past helps us learn new things with little data or effort^44^. Lifelong learning has been proven to leverage the knowledge learned in previous tasks to learn the new task better^45^.

LINGER leverages external data to enhance the inference from single-cell multiome data, incorporating three key steps: training on external bulk data, refining on single-cell data and extracting regulatory information using interpretable artificial intelligence techniques. In our approach, we use a neural network model to fit the expression of TGs, taking as input TF expression and the accessibility of REs. The second layer of the neural network model consists of weighted sums of TFs and REs, forming regulatory modules guided by TF–RE motif matching by incorporating manifold regularization. This leads to the enrichment of TF motifs binding to REs that belong to the same regulatory module. First, we pre-train using external bulk data obtained from the ENCODE project^46^, which contains hundreds of samples covering diverse cellular contexts, referred to as BulkNN.

For refinement on single-cell data, we apply elastic weight consolidation (EWC) loss, using bulk data parameters as a prior. The magnitude of parameter deviation is determined by the Fisher information, which reflects the sensitivity of the loss function to parameter changes. In the Bayesian context, knowledge gained from the bulk data is the prior distribution, forming our initial beliefs about the model parameters. As the model trains on new single-cell data, the posterior distribution is updated, combining the prior knowledge with the likelihood of the new data. EWC regularization encourages the posterior to remain close to the prior, retaining knowledge while adapting, preventing excessive changes and ensuring a more stable learning process^47^. After training the neural network model on single-cell data, we infer the regulatory strength of TF–TG and RE–TG interactions using the Shapley value, which estimates the contribution of each feature for each gene. The TF–RE binding strength is generated by the correlation of TF and RE parameters learned in the second layer (Fig. 1a). LINGER then constructs the cell type-specific and cell-level GRNs based on the general GRN and the cell type-specific profiles (Fig. 1b and Methods).

We will use independent datasets to validate the inference of GRN and then perform several downstream analyses: first, identification of the disease or trait-related cell type, TFs and GRN combining genome-wide association studies (GWAS) data; second, constructing regulon activity on external expression data and identifying driver regulators as differentially active TFs (Fig. 1c).

### LINGER improves the cellular population GRN inference

**Fig. 2:**
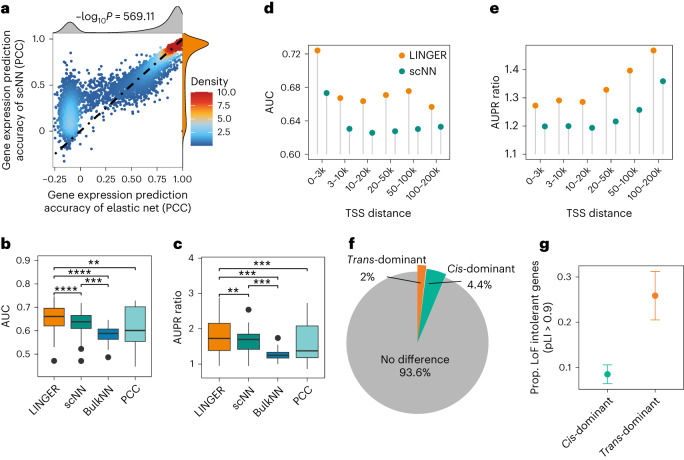
LINGER improves the cellular population GRN inference. **a**, Correlation between predicted and real gene expression, showing higher accuracy for scNN than elastic net. The *x* axis represents the PCC of genes predicted by elastic net and real gene expression across cells, while the *y* axis gives the PCC for scNN. The points represent genes and the color of the points represents the density. The color of distribution in **b**–**e** indicates the different methods: orange, LINGER; gray, elastic net; dark green, scNN; blue, BulkNN; light blue, PCC. Null hypothesis testing results in a *t*-statistic with an effect size of 53.46, df = 15,659, −log_10_*P* = 572.09 and 95% confidence interval of [0.058, 0.063] from a two-sided paired *t*-test. **b**, Boxplot of the performance metric AUC for the predicted *trans*-regulatory strength across all ground truth data. The ground truth data for **b** and **c** are putative targets of TFs from 20 ChIP–seq data points from blood cells (*n* = 20 independent samples). PCC denotes Pearson’s correlation coefficient between the chromatin accessibility of RE and the expression of TG. Note that all boxplots in this study present minima and maxima, the smallest and largest value that is not considered an outlier; center, median; bounds of box, 25th (Q1) to 75th (Q3) percentile; whiskers, 1.5 times the (Q3–Q1). In this study, we use the following convention for symbols indicating statistical significance: ns, *P* > 0.05; *P ≤ 0.05; **P ≤ 0.01; ***P ≤ 0.001; ****P ≤ 0.0001. We hide the ns symbol when displaying significance levels. In detail, *P* = 8.32 × 10^−6^ for LINGER and scNN, *P* = 8.57 × 10^−5^ for LINGER and BulkNN and *P* = 1.24 × 10^−3^ for LINGER and PCC. **c**, Boxplot of the performance metric AUPR ratio for the predicted *trans*-regulatory strength. *P* values in **b** and **c** are from one-sided paired *t*-tests. In detail, *P* = 3.49 × 10^−3^ for LINGER and scNN, *P* = 2.13 × 10^−4^ for LINGER and BulkNN and *P* = 4.53 × 10^−4^ for LINGER and PCC. **d**, AUC for *cis*-regulatory strength inferred by LINGER. The ground truth data for **d** and **e** are the variant-gene links from eQTLGen. We divide RE–TG pairs into different groups based on the distance of the RE from the TSS of TG. **e**, AUPR ratio for *cis*-regulatory strength. **f**, Classification of the *trans*-dominant or *cis*-dominant gene. TFs contribute more to predicting the expression of *trans*-dominant genes, while REs contribute more to *cis*-dominant genes. **g**, Probability of *trans*-dominant and *cis*-dominant being loss-of-function (LoF)-intolerant genes. Points show estimated success probability from binomial distribution, at 0.26 and 0.09 for *trans*-dominant and *cis*-dominant, respectively. *n* = 317 and *n* = 693 independent sample size for *trans*-dominant and *cis*-dominant, respectively. Data are presented as means ± 1.96 × s.d.

To assess the performance of LINGER, we used a public multiome dataset of peripheral blood mononuclear cells (PBMCs) from 10× Genomics (see Methods for details). To investigate whether a linear model is adequate for modeling gene expression or whether a non-linear model is necessary, we conducted a comparative analysis between two models. The first model employs an elastic net to predict the expression of TG by TFs and REs. The second model, single-cell neural network (scNN), is a three-layer neural network model sharing LINGER’s architecture. We assessed the gene expression prediction ability of the two models using fivefold cross-validation. We found that scNN modeled gene expression better than elastic net, with −log_10_*P* = 572.09, especially for those substantial proportions of genes that show negative Pearson’s correlation coefficient (PCC) in elastic net predictions (−log_10_*P* = 1,060.17; Fig. 2a). This demonstrates that the three-layer neural network model scNN outperforms the elastic net model in predicting gene expression.

To show the utility and effectiveness of integrating external bulk data, we compared LINGER to scNN, BulkNN and PCC. To evaluate the performance of *trans*-regulatory strength, we collected putative targets of TFs from chromatin immunoprecipitation followed by sequencing (ChIP–seq) data using a systematical standard (Methods) and, in total, obtained 20 data sets in blood cells as ground truth^48^ (Supplementary Table 1). For each ground truth, we calculated the area under the receiver operating characteristic curve (AUC) and the area under the precision–recall curve (AUPR) ratio (see Methods) by sliding the *trans*-regulatory predictions. Results show that scNN performs better than PCC and BulkNN. Compared to other methods, LINGER performs better, with a significantly higher AUC (Fig. 2b) and AUPR ratio (Fig. 2c) across all ground truth data.

**Extended Data Fig. 1:**
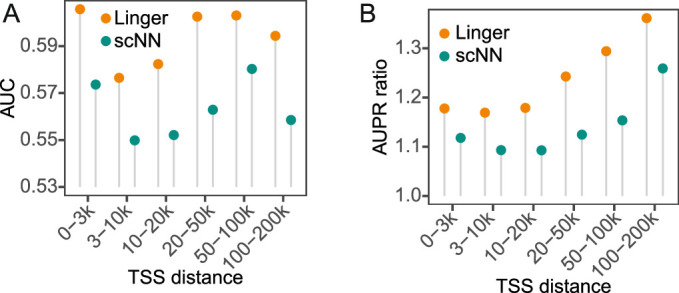
Assessing the performance of *cis*-regulatory strength inferred by LINGER taking eQTL data for GTEx as ground truth. **A**. AUC for *cis*-regulatory strength inferred by LINGER. The ground truth for **A** and **B** is the variant-gene links from GTEx. We divide RE-TG pairs into different groups based on the distance of RE and the TSS of TG. **B**. AUPR ratio for *cis*-regulatory strength.

To validate the *cis*-regulatory inference of LINGER, we calculated the consistency of the *cis*-regulatory coefficients with expression quantitative trait loci (eQTL) studies that link genotype variants to their TGs. We downloaded variant-gene links defined by eQTL in whole blood from GTEx^49^ and eQTLGen^50^ (Supplementary Table 2) as ground truth. As the distance between RE and TG is important for the prediction, we divided RE–TG pairs into different distance groups. LINGER achieved a higher AUC and AUPR ratio than scNN in all different distance groups in eQTLGen (Fig. 2d,e) as well as GTEx (Extended Data Fig. 1a,b). The above results show that LINGER improves the *cis*-regulatory and *trans*-regulatory strength inference by leveraging external data.

We next sought to investigate the dominant regulation for genes; that is, whether a gene is mainly regulated by *cis*-regulation or *trans*-regulation. To shed light on this question, we compared the average of *cis*-regulatory and *trans*-regulatory strength Shapley values by a two-sided unpaired *t*-test and performed Bonferroni *P* value correction. Our findings reveal that most genes exhibit no significant difference in *cis*-regulation and *trans*-regulation dominance. Specifically, 4.37% of genes are *cis*-regulation dominant, while 2.00% are *trans*-regulation dominant (Fig. 2f). To discern evolutionary distinctions between *trans*-dominant and *cis*-dominant genes, we compared their strength of selection using pLI, which is an estimate of the ‘probability of being loss of function intolerant’^51^. We observed that the percentage of selectively constrained genes with high pLI (>0.9) in the *trans*-dominant group was approximately three times higher than that in the *cis*-dominant group (Fig. 2g). A previous study found that disease-associated genes from GWAS were enriched in selectively constrained genes, while eQTL genes were depleted in selectively constrained genes^52^. These observations highlight the importance of the *trans*-regulatory network in understanding complex diseases. Functional enrichment analysis^53^ shows that the *cis*-regulatory dominant genes were significantly enriched in 38 GTEx aging signatures (Supplementary Table 3), which aligns with the conclusion that chromatin accessibility alterations occur in age-related macular degeneration^54^.

**Extended Data Fig. 2:**
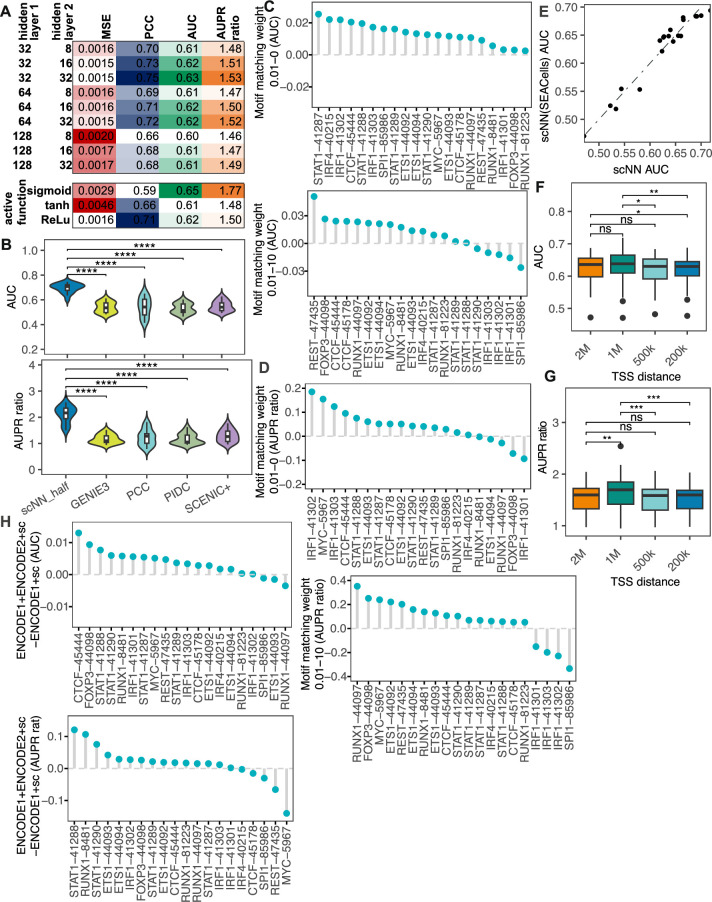
Parameter sensitivity. **A**. Sensitivity of neural network structure and active function. **B**. Violin plot of AUC and AUPR ratio values of *trans*-regulatory potential performance across diverse TFs and cell types (n=20 independent sample). One-sided paired t-test result in -log_10_P-value 10.73, 7.11, 10.85, and 9.61 compared with GENIE3, PCC, PIDC, and SCENIC+ in terms of AUC, respectively. For AUPR ratio, -log_10_P-values are 8.94, 7.03, 8.48, and 7.57, respectively. **C**, **D**. Bar plot of AUC and AUPR ratio difference of different motif matching weight. The upper and lower figures refer to the difference in weight 0.01 to 0 and 0.01 to 10. The x-axis of **C**, **D**, and **H** refers to the ground truth data named by the TF name and Cistrome database ID. **E**. Scatter plot of AUC of original metacells and SEACells metacells as input. Each point refers to each ChIP-seq ground truth data. **F, G**. Box plot of AUPR ratio and AUC of defining regulatory element within different TSS distances from 200 Kb to 2 Mb (n = 20 independent sample). Two-sided paired t-test result in p-value 0.055(2 Mb and 1 Mb), 0.088(2 Mb and 500 Kb), 0.028(2 Mb and 200 Kb), 0.025(1 Mb and 500 Kb), 0.0056(1 Mb and 200 Kb), and 0.70(500 Kb and 200 Kb) in terms of AUC. For AUPR ratio, p-values are 0.0017(2 Mb and 1 Mb), 0.093(2 Mb and 500 Kb), 0.12(2 Mb and 200 Kb), 0.00048(1 Mb and 500 Kb), 0.00075(1 Mb and 200 Kb), and 0.64(500 Kb and 200 Kb). **H**. Bar plot of AUC and AUPR ratio difference of two rounds pre-train and single round pre-train.

To gain an understanding of parameter sensitivity, we systematically evaluated the effects of TF–RE motif matching, *cis*-REs transcription start site (TSS) distance, activation function, number of nodes in hidden layers and metacell-generating method on the scNN. Note that the sigmoid activation function would not improve the gene expression prediction but would improve the GRN inference (Extended Data Fig. 2a). Using motif matching information by manifold regularization loss properly by setting the weight will improve the performance. Compared to 0, weight 0.01 improved the performance on 100% (Extended Data Fig. 2c) and 80% (Extended Data Fig. 2d) of ground truth data based on the AUC and AUPR ratio, respectively. The performance of weight 10 decreases compared to 0.01 (Extended Data Fig. 2c,d). To verify the robustness of our method to alternative metacell-generation approaches (see ‘PBMC 10× data’ in Methods), we used metacells generated by the SEACells as a substitute for our original metacells. There were no significant differences in the performance between SEACells metacells and our original metacells (two-sided paired *t*-test, *P* = 0.89; Extended Data Fig. 2e). Using REs within 1 Mb is the best across 200 kb, 500 kb, 1 Mb and 2 Mb (Extended Data Fig. 2f,g).

We evaluated LINGER’s capability for lifelong learning by leveraging additional data sources. We split the ENCODE data into two batches (ENCODE1, ENCODE2) and applied two rounds of pre-training, then trained on PBMCs single-cell multiome data (ENCODE1+ENCODE2+sc). We compared the results with those obtained by using one batch of ENCODE data as pre-training (ENCODE1+sc). Extended Data Fig. 2h shows that compared to single pre-training, the addition of the second round of pre-training improved the performance of TF–TG inference for 85.5% (17 out of 20) and 75% (15 out of 20) of ChIP–seq data based on the AUC and AUPR ratio, respectively. This validates LINGER’s capability for continuous refinement through incremental learning from diverse datasets.

### LINGER improves the cell type-specific GRN inference

We evaluated the cell type-specific GRN inference (Methods) of LINGER in PBMCs sc-multiome data as well as an in-silico mixture of H1, BJ, GM12878 and K562 cell lines from single-nucleus chromatin accessibility and mRNA expression sequencing (SNARE-seq) data^55^. To assess TF–RE binding prediction, we used ChIP–seq data as ground truth, including 20 TFs from four cell types within the blood and 33 TFs from the H1 cell line^48^ (Supplementary Table 4). The putative target of TF from the ChIP–seq data serves as ground truth for the *trans*-regulatory potential. For the *cis*-regulatory potential, we incorporated promoter-capture Hi-C data of three primary blood cell types (Supplementary Table 5)^56^ and single-cell eQTL^57^, including six immune cell types as ground truth for PBMCs.

**Fig. 3:**
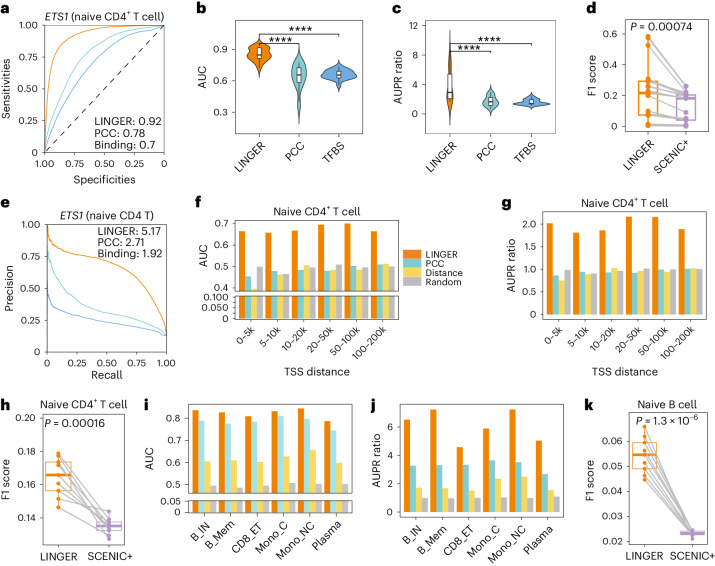
Systematic benchmarking of cell type-specific TF–RE binding potential and *cis*-regulatory potential performance. **a**,**e**, Receiver operating characteristic curve and precision–recall curve of binding potential for *ETS1* in naive CD4 T cells. The ground truth for **a** and **e** is the ChIP–seq data of *ETS1* in naive CD4^+^ T cells. The color in **a**–**e** represents the different methods used to predict TF–RE regulation. Orange, LINGER; green, PCC between the expression of TF and the chromatin accessibility of RE; blue, motif binding affinity of TF to RE. **b**,**c**, Violin plot of the AUC and AUPR ratio values of binding potential across diverse TFs and cell types. The ground truth is the ChIP–seq data for 20 TFs from different cell types in blood. The original data is in Supplementary Table 6. The null hypothesis testing in **b**, comparing the AUC of LINGER with PCC and binding, results in *t*-statistics (one-sided paired *t*-test) with effect size, 8.99; df, 19; *P* = 1.42 × 10^−8^, 95% confidence intervals, [0.17, Inf] and effect size, 18.25; df, 19; *P* = 8.34 × 10^−14^; 95% confidence intervals, [0.17, Inf], respectively. The null hypothesis testing in **c**, comparing the AUPR ratio of LINGER with PCC and binding, results in t-statistics (one-sided paired *t*-test) with effect size, 4.65; df, 19; *P* = 8.72 × 10^−5^; 95% confidence intervals, [1.31, Inf] and effect size, 5.44, df, 19; *P* = 1.49 × 10^−5^; 95% confidence intervals, [1.51, Inf], respectively. **d**, The performance metrics F1 score of binding potential. Each point represents ground truth data (*n* = 20 independent samples). The *P* values for **d**, **h** and **k** are based on one-sided paired *t*-tests. **f**,**g**, AUC and AUPR ratio of *cis*-regulatory potential in naive CD4^+^ cells. The ground truth for **f**–**h** is promoter-capture Hi-C data. RE–TG pairs are divided into six distance groups ranging from 0–5 kb to 100–200 kb. PCC is calculated between the expression of TG and the chromatin accessibility of RE. Distance denotes the decay function of the distance to the TSS. Random denotes the uniform distribution from 0 to 1. **h**, F1 score of *cis*-regulatory in naive CD4^+^ cells for LINGER and SCENIC+ (*n* = 9 independent samples). **i**,**j**, AUC and AUPR ratio of *cis*-regulatory potential. The ground truth is eQTL data from six immune cell types. **k**, F1 score of *cis*-regulatory potential in naive B cells. The ground truth is eQTL data from naive B cells (*n* = 9 independent samples). This figure corresponds to the PBMC data.

**Extended Data Fig. 3:**
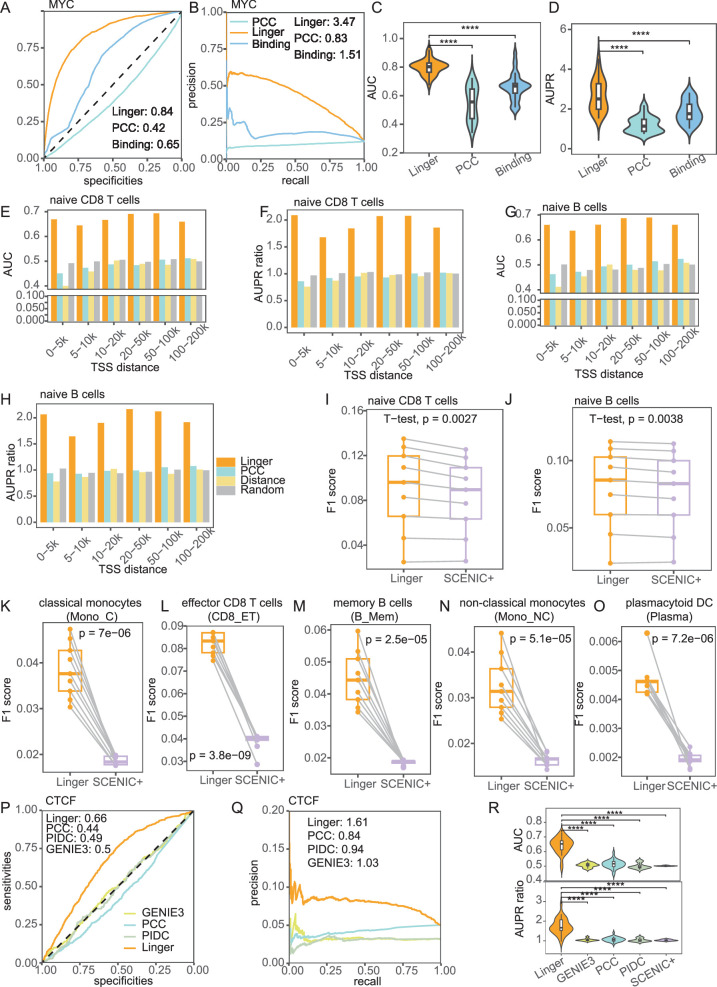
Systematic benchmarking of cell type-specific GRN. **A, B**. ROC curve and PR curve of binding potential for MYC in H1 cell line. The ground truth for **A** to **D** is the ChIP-seq data of MYC in the H1 cell line. The color in **A** to **D** represents the different competitors to predict TF-RE regulation. Orange represents LINGER, green represents PCC between the expression of TF and the chromatin accessibility of RE, and blue represents motif binding affinity of TF to RE. **C, D**. Violin plot of AUC and AUPR ratio values of binding potential across diverse TFs. The ground truth is ChIP-seq data for 33 TFs (n=33 independent sample). One-sided paired t-test is performed to test whether there is significant difference. In **C**, -log_10_P-values are 11.36 and 12.27 compared with PCC and TFBS, respectively. In **D**, -log P-values are 6.21 and 5.18, respectively. **E**, **F**. AUC and AUPR ratio of *cis*-regulatory potential in naïve CD8 T cells. The ground truth for **E** to **J** is promoter capture HiC data. RE-TG pairs are divided into six distance groups ranging from 0-5k to 100-200 kb. PCC is calculated between the expression of TG and the chromatin accessibility of RE. Distance denotes the decay function of the distance to the TSS. Random denotes the uniform distribution. **G**, **H**. AUC and AUPR ratio of *cis*-regulatory potential in naïve B cells. **I**, **J**. F1 score of *cis*-regulatory in naïve CD8 T cells and naïve B cells for LINGER and SCENIC+. P-values are from one-sided paired t-test with n=9 independent sample. **K** to **O**, F1 score of *cis*-regulatory potential in classical monocytes, effector CD8 T cells, memory B cells, non-classical monocytes, and plasmacytoid DC cells for LINGER and SCENIC+. The ground truth is eQTL data (n=9 independent sample). P-values are from one-sided paired t-test. **P**, **Q**. ROC curve and PR curve of *trans*-regulatory potential inference of CTCF in H1 cell line. The ground truth of **P** to **R** is putative targets of TFs from ChIP-seq data in the H1 cell line. **R** Violin plot of AUC and AUPR ratio values of *trans*-regulatory potential performance across diverse TFs in H1 cell line (n=33 independent sample). One-sided unpaired t-test result in -log_10_P-value 15.89, 15.64, 16.36, and 15.54 compared with GENIE3, PCC, PIDC, and SCENIC+ in terms of AUC, respectively. For AUPR ratio, -log_10_P-values are 11.01, 10.64, 11.20, and 11.17, respectively.

To assess the TF–RE binding potential, we compared our method with TF–RE correlation (PCC) and motif binding affinity. For example, in naive CD4 T cells, LINGER achieves an AUC of 0.92 and an AUPR ratio of 5.17 for *ETS1*, which is an improvement over PCC (AUC, 0.78; AUPR ratio, 2.71) and motif binding affinity (AUC, 0.70; AUPR ratio, 1.92) (Fig. 3a,e). For binding sites of *MYC* in the H1 cell line, LINGER outperforms PCC and motif binding affinity-based predictions (Extended Data Fig. 3a,b). For all 20 TFs in PBMCs, LINGER consistently exhibits the highest AUC and AUPR ratios, and the overall distributions are significantly higher than others in PBMCs (*P* ≤ 8.72 × 10^−5^; Fig. 3b,c and Supplementary Table 6). LINGER also outperforms other methods for H1 data (*P* ≤ 6.68 × 10^−6^; Extended Data Fig. 3c,d). Furthermore, we compared LINGER with a state-of-the-art method, SCENIC+^42^, which predicts TF–RE pairs from multiome single-cell data. Given that SCENIC+ does not provide a continuous score for all REs, we used the F1 score as a measure of accuracy. Fig. 3d shows that LINGER performs better for all 20 TFs binding site predictions.

To assess the *cis*-regulatory potential, we compared LINGER with four baseline methods, including distance-based methods, RE–TG correlation (PCC), random predictions, and SCENIC+. We divided RE–TG pairs of Hi-C data into six distance groups ranging from 0–5 kb to 100–200 kb. In naive CD4 T cells, LINGER achieves AUC ranging from 0.66 to 0.70 (Fig. 3f) and AUPR ratio ranging from 1.81 to 2.16 (Fig. 3g) across all distance groups, while other methods are close to random. In other cell types, LINGER exhibits consistent superiority over the baseline methods (Extended Data Fig. 3e–h). All eQTL pairs were considered positive labels owing to the insufficient pairs available for division into distance groups. In all cell types, the AUC and AUPR ratio of LINGER are higher than the baseline methods (Fig. 3i,j). We also compared our method with SCENIC+, which outputs predicted RE–TG pairs without importance scores. We selected the same number of top-ranking RE–TG pairs and calculated the F1 score using nine cutoffs corresponding to quantiles ranging from the 10th to the 90th percentile. As a result, LINGER attains significantly higher F1 scores than SCENIC+ in all cell types (Fig. 3h and Extended Data Fig. 3i,j) based on Hi-C data. Taking eQTL as ground truth, the F1 score of LINGER is significantly higher than SCENIC+ (Fig. 3k) and other cell types (Extended Data Fig. 3k–o).

**Fig. 4:**
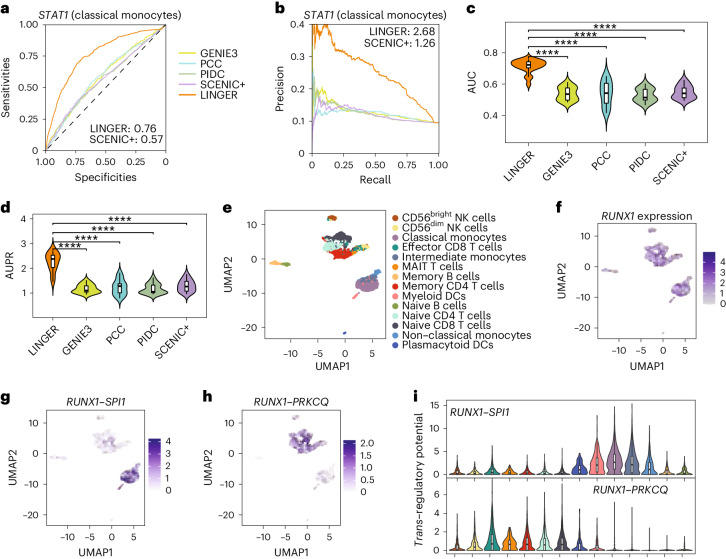
Systematic benchmarking of cell type-specific *trans*-regulatory potential performance. **a**,**b**, Receiver operating characteristic curve and precision–recall curve of *trans*-regulatory potential inference of *STAT1* in classical monocytes. The ground truth data in **a**–**d** are putative targets of TFs from ChIP–seq data for the corresponding cell types in PBMCs. **c**,**d**, Violin plot of AUC and AUPR ratio values of *trans*-regulatory potential performance across diverse TFs and cell types. The original data is in Supplementary Table 7. The sample size for the one-sided paired *t*-test is 20. For **c**, −log_10_(*P* values) are 11.12, 7.72, 11,13 and 10.17 for GENIE3, PCC, PIDC and SCENIC+, respectively. For **d**, −log_10_(*P* values) are 9.59, 8.02, 9.22 and 8.47, respectively. **e**, Uniform manifold approximation and projection (UMAP) of PBMCs including 14 cell types. NK cells, natural killer cells; MAIT, mucosal-associated invariant T cells; DCs; dendritic cells. **f**, UMAP of *RUNX1* expression across PBMCs. **g**, UMAP of cell level *trans*-regulatory potential for *RUNX1*(TF)*–SPI1*(TG) across PBMCs. **h**, UMAP of cell level *trans*-regulatory potential for *RUNX1*(TF)*–PRKCQ*(TG) across PBMCs. **i**, Violin plot of cell level *trans*-regulatory potential from different cell types. The sample size for each boxplot is the number of cells of each cell type, ranging from 98 to 1,848. This figure corresponds to the PBMCs.

**Extended Data Fig. 4:**
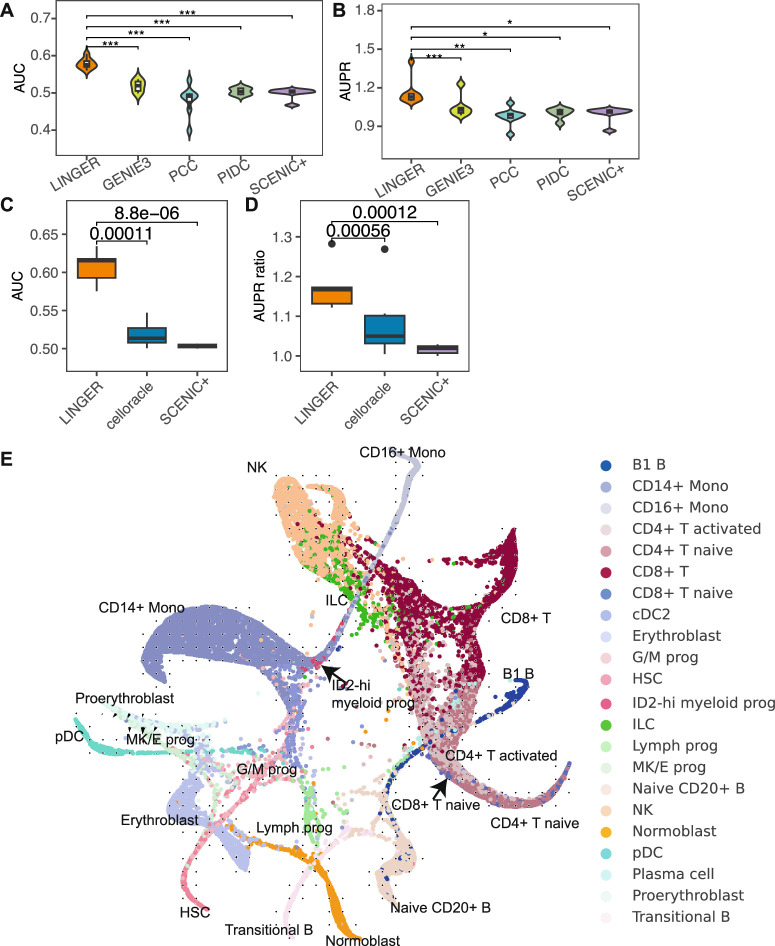
*In silico* perturbation. **A**, **B**. Violin plot of AUC and AUPR ratio values of *trans*-regulatory potential performance across diverse TFs and cell types for PBMCs. The ground truth of **A** to **D** is 8 experimental perturbation data from KnockTF database (n=8 independent sample). One-sided paired t-test are performed to test the difference. For AUC, -log_10_P-values are 3.74, 3.43, 3.64, and 3.86 compared with GENIE3, PCC, PIDC, and SCENIC+, respectively. For AUPR ratio, -log_10_P-values are 3.36, 2.14, 1.69 and 1.80, respectively. **C**, **D**. Box plot of AUC and AUPR ratio values of in silico perturbation predicted target gene. P-values are from one-sided paired t-test with 8 independent samples. **E**. Differentiation behavior prediction on BMMC data after knocking out GATA1.

To evaluate the accuracy of *trans*-regulatory potential, we chose GENIE3 (ref. ^15^) and PIDC^21^ for comparison based on the benchmarking literature of GRN inference from single-cell data^39^ that we chose in previous work^58^ (see Methods). In addition, we compared LINGER with PCC and SCENIC+. For *STAT1* in classical monocytes, LINGER improves the prediction performance, as evidenced by an AUC of 0.76 versus 0.57–0.59 and an AUPR ratio of 2.60 versus 1.26–1.36 (Fig. 4a,b). A similar improvement is observed for *CTCF* in H1 (Extended Data Fig. 3p,q). The average AUPR ratio across ground truth datasets for other methods was 1.17–1.29, 0.17–0.29 units above random prediction, whereas LINGER achieves 1.25 units above random prediction, indicating a fourfold to sevenfold relative increase (Fig. 4d). Overall, LINGER consistently performs better than other methods for all 20 TFs in PBMCs, with a significantly higher AUC and AUPR ratio (*P* ≤ 9.49 × 10^−9^; Fig. 4c,d and Supplementary Table 7). LINGER outperforms other competitors in the H1 cell line (*P*  ≤ 3.00 × 10^−8^; Extended Data Fig. 3r). Unlike GENIE3 and PIDC, which solely use scRNA-seq data, our method effectively doubles the cell data by integrating both scRNA-seq and scATAC-seq. For a fairer comparison, we removed pre-training and used only half as many cells as input (scNN_half). Comparing to other competitors showed that scNN_half continued to significantly outperform all other methods (Extended Data Fig. 2b). We also evaluated cell type-specific *trans*-regulatory potential to predict direct differentially expressed genes (DEGs) under perturbation of the TF, using perturbation experiment data as ground truth. We collected eight datasets for PBMCs (Supplementary Table 8) from the KnockTF database^59^. Extended Data Fig. 4a,b shows that LINGER outperforms all other methods (*P* ≤ 3.72 × 10^−4^).

The rationale for constructing a single-cell-level GRN is the same as a cell type-specific GRN, replacing the cell type-specific term with the single-cell term (Methods). We show the result of *trans*-regulation, taking *RUNX1* as an example. *RUNX1* is critical for establishing definitive hematopoiesis^60^ and expresses at high levels in almost all PBMC cell types (Fig. 4e,f). *RUNX1* regulates *SPI1* in monocytes (classical, non-classical and intermediate) and myeloid dendritic cells (Fig. 4g,i), while regulates *PRKCQ* in CD56^dim^ natural killer cells, effector CD8 T cells, mucosal-associated invariant T cells, memory CD4 T cells, naive CD4 T cells and naive CD8 T cells (Fig. 4h,i). This example illustrates the capability of LINGER to visualize gene regulation at the single-cell level.

### LINGER reveals the regulatory landscape of GWAS traits

GWASs have identified thousands of disease variants, but the active cells and functions involving variant-regulated genes remain largely unknown^61^. We integrate GWAS summary statistics and cell type-specific GRN to identify the relevant cell types, key TFs and sub-GRN (Methods). We define a trait regulation score for TFs in each cell type, measuring the enrichment of GWAS genes downstream of TFs. In trait-relevant cell types, TFs with high trait regulation scores should be expressed to perform their function. We identify the trait-relevant cell types by assessing the concordance between TF expression and the trait regulation score.

**Fig. 5:**
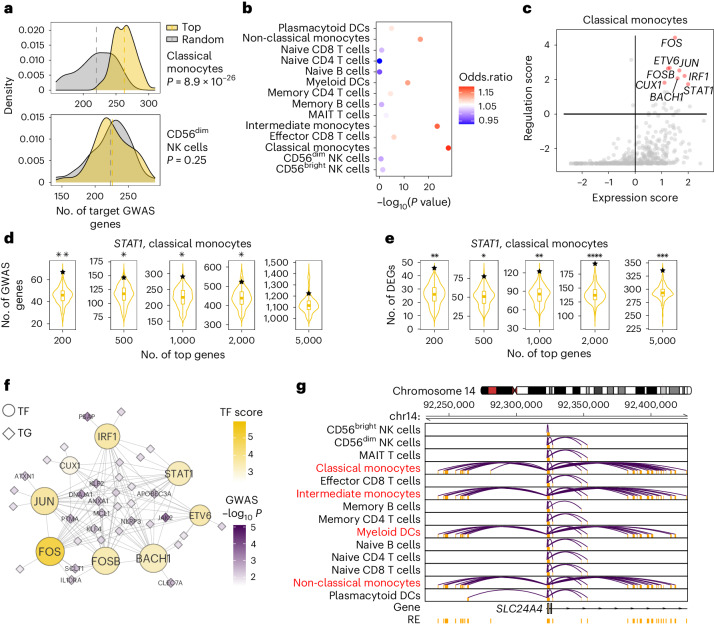
Elucidating GWAS traits through LINGER-inferred regulatory landscape. **a**, Distribution of the number of TGs for top expression TFs and randomly selected TFs in classical monocytes (top) and CD56^dim^ NK cells (bottom). The 100 top-expression TFs and 100 randomly selected TFs are used to generate the distribution. **b**, Enrichment of IBD GWAS to cell types in PBMCs. The color of the bubbles corresponds to the odds ratio of the number of TGs between top expression and randomly selected TFs. The *x* axis is the −log_10_(*P* value) from the one-sided unpaired *t*-test for the number of TGs between top expression and randomly selected TFs. **c**, Key IBD-associated regulators in classical monocytes. The *x* axis is the *z*-score of the expression of TFs across all TFs. The *y* axis is the regulation score of TFs. The TFs in red are the top-ranked TFs according to the summation of the expression level and regulation score. **d**, Enrichment of GWAS IBD genes among *STAT1* targets in classical monocytes. The violin plot is generated by randomly choosing 1,000 TFs; the number of overlapping genes for *STAT1* is marked by a star. The different violin plots correspond to taking the top 200–5,000 genes as the TG for each TF, respectively. **e**, Enrichment of DEGs between inflamed biopsies and non-inflamed biopsies among *STAT1* targets in classical monocytes. The details are the same as in **d**. **f**, Sub-network of IBD-relevant TFs from classical monocytes *trans*-regulatory network. The size of the TF or TG nodes corresponds to their degree in the network. The color of TF denotes the trait-relevant score, and the color of TG denotes the −log_10_(*P* value) of GWAS SNP assigned to the gene. **g**, *Cis*-regulatory network at locus around *SLC24A4*. The interaction denotes significant RE–TG links, and we use the location of the promoter to represent the gene.

In our specific study on inflammatory bowel disease (IBD), we collected the risk loci based on a GWAS meta-analysis of about 330,000 individuals from the NHGRI-EBI GWAS catalog^62^ for study GCST90225550^63^. Figure 5a shows that in classical monocytes, trait regulation scores for the top-expressed TF are significantly higher than randomly selected TFs (*P* = 8.9 × 10^−29^, one-sided unpaired *t*-test), while there is no significant difference for non-relevant cell types such as CD56^dim^ natural killer cells. The most relevant cell types in PBMCs are monocytes and myeloid dendritic cells (Fig. 5b). These findings align with previous studies linking monocytes to the pathogenesis of IBD^64,65^.

We next identified key TFs by the sum of the expression level and trait regulation score. Figure 5c lists the top eight candidate TFs in classical monocytes. These TFs have been previously reported to be associated with IBD in the literature. *FOS* can increase the risk of recurrence of IBD^66^; one variant identified in the IBD cohort is located at the exon of *ETV6*; *IRF1* and *ETV6* are key TFs with activity differences in IBD^67^; genes *FOS*, *FOSB* and *JUN* encode potent mediators of IBD^68^; *CUX1* is induced in IBD^69^; and *STAT1* epigenetically contribute to the pathogenesis of IBD^70^.

To investigate the downstream targets of key TFs, we chose *STAT1* as an example. Among the top 200 TGs regulated by *STAT1* in classical monocytes, 67 of them overlap with the GWAS genes, which is statistically significant with a P value of less than 0.01 based on a background distribution from a random selection of TFs (one-sided bootstrap hypothesis testing). The numbers of overlapped TGs are all significant for the top 500, 1,000, 2,000 and 5,000 TGs (Fig. 5d). Apart from GWAS-relevant genes, we collected the DEGs between inflamed biopsies and non-inflamed biopsies^71^ and we found that these DEGs significantly overlapped with the top-ranked TGs of *STAT1* (one-sided bootstrap hypothesis testing; Fig. 5e). The lack of significant overlap between DEGs and GWAS genes (*P* = 0.15, two-sided Fisher’s exact test) but the significant overlap of both DEGs and GWAS with the top-ranked TGs of *STAT1* indicates the robustness and unbiased nature of our method.

Finally, we extracted the sub-network of the eight candidate TFs from the classical monocyte *trans*-regulatory network for IBD (Fig. 5f). We also observed that the *cis*-regulatory network of *SLC24A4* (Fig. 5g), 46 kb from a risk single nucleotide polymorphism (SNP) rs11626366 (*P* = 7.4 × 10^−3^), is specifically dense in the IBD-relevant cell types, which shows the complex regulatory landscape of disease genes across different cell types.

### Identify driver regulators based on transcription profiles

Researchers often identify DEGs between cases and controls using bulk or single-cell expression data, but the underlying regulatory drivers remain elusive. TF activity, focusing on the DNA-binding component of TF proteins, is a more reliable metric than mRNA for identifying driver regulators. One feasible approach is to estimate TF activity based on the expression patterns of downstream TGs, which necessitates the availability of an accurate GRN. Assuming that the GRN structure is consistent for the same cell type across individuals, we employed LINGER-inferred GRNs from single-cell multiome data of a single individual to estimate the TF activity of other individuals using gene expression data alone from the same cell type. By comparing TF activity between cases and controls, we identified driver regulators. This approach is valuable, as it leverages limited single-cell multiome data to estimate TF activity in multiple individuals using only gene expression data (see Methods). We present two illustrative examples showcasing its utility.

**Fig. 6:**
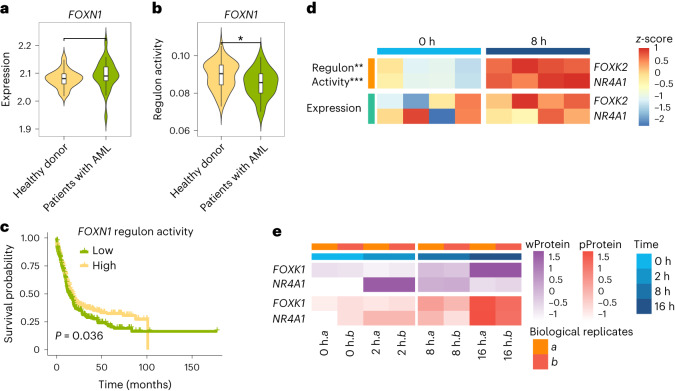
Driver regulator identification. **a**, Violin plot of *FOXN1* expression across healthy donors (*n* = 38 independent samples) and patients with AML (*n* = 26 independent samples), respectively. There is no significant difference in the mean expression (two-sided unpaired *t*-test). **b**, Violin plot of regulon activity of *FOXN1* across healthy donors (*n* = 38 independent samples) and patients with AML (*n* = 26 independent samples), respectively (two-sided unpaired *t*-test, *P* = 0.035). **c**, AML survival by the regulon activity of *FOXN1* (*P* value is from a two-sided log-rank test). **d**, The heatmap of regulon activity and gene expression in response to TCR stimulation at 0 h and 8 h. Two-sided unpaired *t*-test for the difference in regulon activity, *P* = 0.0057 and *P* = 0.00081 for *FOXK1* and *NR4A1*, respectively; the *P* value for gene expression is >0.05. Heatmap is scaled by row. **e**, Heatmap of whole protein (wProtein) and phosphoproteomics (pProtein) expression in response to TCR stimulation at 0 h, 2 h, 8 h and 16 h. There are two biological replicates, represented by *a* and *b*. The wProtein and pProtein expression of *FOXK1* and *NR4A1* is higher at 8 h than at 0 h. The heatmap is scaled by row.

Example 1: We collected the bulk gene expression data from 26 patients with acute myeloid leukemia (AML) and 38 healthy donors^72^. We calculated the TF activity for these samples based on the LINGER-inferred cell population GRN from PBMCs and found that *FOXN1* is significantly less active in patients with AML than in healthy donors, and it is not differentially expressed (Fig. 6a,b). In addition, we calculated the TF activity of the transcriptome profile (bulk RNA-seq data) of 671 individuals with AML^73^ and performed survival analysis, which indicated that individuals with high *FOXN1* activity level tend to have a higher survival probability (Fig. 6c). Furthermore, *FOXN1* has been reported as a tumor suppressor^74^.

Example 2: We also present an example of the naive CD4^+^ T cell response upon T cell receptor (TCR) stimulation^75^, which induces T cell differentiation into various effector cells and activates T lymphocytes. We calculated the TF activity based on the GRN of naive CD4^+^ T cells and identified differentially active regulators in response to TCR stimulation at 8 h versus 0 h. *FOXK2* and *NR4A1* are activated at 8 h based on regulon activity (Fig. 6d), which is consistent with the whole proteomics and phosphoproteomics data (Fig. 6e)^76^. Other studies have also shown that *FOXK2* affects the activation of T lymphocytes^77,78^ and revealed the essential roles of *NR4A1* in regulatory T cell differentiation^79,80^, suggesting that the identified TFs have important roles in naive CD4^+^ T cell response upon TCR stimulation. However, *FOXK2* and *NR4A1* show no significant differences in expression at 8 h versus 0 h (Fig. 6d).

### In silico perturbation

We performed in silico perturbation to predict the gene expression after knocking out TFs. To do so, we changed the expression of an individual TF or combinations of TFs to zero and used the predicted gene expression as the in silico perturbation gene expression. We used the expression difference before and after in silico perturbation to infer the TG. To assess the performance of the prediction, we collected perturbation data for eight TFs in blood cells from the KnockTF^59^ database (Supplementary Table 8) as ground truth. We performed the in silico individual TF perturbation of the eight TFs using LINGER. As a comparison, we performed identical computational perturbation experiments using the CellOracle^81^ and SCENIC+^42^ methods. The results, shown in Extended Data Fig. 4c,d, demonstrate that LINGER is more accurate than the alternative approaches (*P*  ≤ 3.72 × 10^−4^).

To assess LINGER’s capability to infer differentiation behavior, we leveraged CellOracle^81^ as a downstream analytical tool. We used the LINGER-inferred GRN as an input to CellOracle. This allowed us to investigate the capacity of LINGER-derived networks to recapitulate differentiation responses. Examining bone marrow mononuclear cell data^82^, which contains progenitor populations, we performed an in silico knockout of *GATA1*, a known key regulator of erythroid and megakaryocytic differentiation^83^. CellOracle predictions based on the LINGER GRN showed that *GATA1* knockout shifted proerythroblasts to a megakaryocytic or erythroid progenitor state (Extended Data Fig. 4e), consistent with the functional role of *GATA1* in inhibiting erythroblast maturation. These results demonstrate that LINGER can not only predict gene expression under perturbation but also enable downstream characterizations of differentiation trajectories through integration with complementary analytical frameworks like CellOracle.

## Conclusions and discussions

LINGER is an neural network-based method that infers GRNs from paired single-cell multiomic data by incorporating bulk datasets and knowledge of TF–RE motif matching. Compared to existing tools, LINGER achieves substantially higher GRN inference accuracy. A key innovation is lifelong machine learning to leverage diverse cellular contexts, continually updating the model as new data emerge. This addresses historic challenges from limited single-cell datasets and vast parameter spaces hindering complex model fitting. LINGER’s lifelong learning approach has the advantage of pre-training on bulk collections, allowing users to easily retrain the model for their own studies while capitalizing on publicly available resources without direct access. Traditionally, GRN inference performance is assessed by gene expression prediction. However, the use of lifelong learning to leverage external data does not lead to improved gene expression prediction but does improve the GRN inference. This finding challenges the traditional strategy of evaluating GRN inference solely based on gene expression prediction and highlights the importance of considering the overall network structure and regulatory interactions.

The lifelong learning mechanism will encourage the model to retain prior knowledge from the bulk data when adapting to the new single-cell data. It is a tradeoff between retaining prior knowledge and fitting new data. The flexibility of the variation in prior knowledge is not constrained when fitting the new data. The extent to which the final result deviates from the prior knowledge depends on the loss incurred in fitting the new data. LINGER will learn this tradeoff automatically to obtain a maximized usage of the information from both datasets.

## Methods

### GRN inference by lifelong learning

LINGER is a computational framework to infer GRNs—pairwise regulation among TGs, REs and TFs—from single-cell multiome data. Overall, LINGER predicts gene expression by the TF expression and chromatin accessibility of REs based on neural network models. The contribution of each feature is estimated by the Shapley value of the neural network models, enabling the inference of the GRNs. To capture key information from the majority of tissue lineages, LINGER uses lifelong machine learning (continuous learning). Moreover, LINGER integrates motif binding data by incorporating a manifold regularization into the loss function.

The inputs for full training of LINGER are external bulk and single-cell paired gene expression and chromatin accessibility data. However, we provided a bulk data pre-trained LINGER model so that users can retrain it for their own single-cell data without accessing external bulk data. We collected paired bulk data—gene expression profiles and chromatin accessibility matrices—from 201 samples from diverse cellular contexts^84^ from the ENCODE project^46^. Single-cell data are raw count matrices of multiome single-cell data (gene counts for RNA-seq and RE counts for ATAC-seq). LINGER trains individual models for each gene using a neural network architecture that includes an input layer and two fully connected hidden layers. The input layer has dimensions equal to the number of features, containing all TFs and REs within 1 Mb of the TSS for the gene to be predicted. The first hidden layer has 64 neurons with rectified linear unit activation that can capture regulatory modules, each of which contains multiple TFs and REs. These regulatory modules are characterized by enriched motifs of the TFs on the corresponding REs. The second hidden layer has 16 neurons with rectified linear unit activation. The output layer is a single neuron, which outputs a real value for gene expression prediction.

We first construct neural network models based on bulk data, using the same architecture described above. We extract the TF expression matrix ${\widetilde{E}}_{{\rm{TF}}}\in {{\mathbb{R}}}^{{N}_{{\rm{TF}}}\times {N}_{b}}$ from the bulk gene expression matrix $\widetilde{E}\in {{\mathbb{R}}}^{{N}_{{\rm{TG}}}\times {N}_{b}}$, with ${N}_{{\rm{TG}}}$ representing the number of genes, ${N}_{{\rm{TF}}}$ representing the number of TFs and ${N}_{b}$ representing the number of tissues. The loss function consists of mean squared error (MSE) and L1 regularization, which, for the *i*^th^ gene is:

$${ {\mathcal L} }_{\rm{BULK}}\left({\tilde{E}}_{\rm{TF}},{\tilde{O}}^{(i)},{\tilde{E}}_{i,\cdot },{\theta }_{b}^{(i)}\right)=\frac{1}{{N}_{b}}\mathop{\sum }\limits_{n=1}^{{N}_{b}}{\left(\;f\left({({\tilde{E}}_{\rm{TF}})}_{\cdot ,n},{\tilde{O}}_{\cdot ,n}^{(i)},{\theta }_{b}^{(i)}\right)-{\tilde{E}}_{in}\right)}^{2}{+}{\lambda }_{0}{\Vert {\theta }_{b}^{(i)}\Vert }_{1}$$

where $\widetilde{O}\in {{\mathbb{R}}}^{{N}_{{\rm{RE}}}^{(i)}\times {N}_{b}}$ represents the chromatin accessibility matrix, with ${N}_{{\rm{RE}}}^{(i)}$ REs within 1 Mb of the TSS of the *i*^th^ gene, and $f\left({\left({\widetilde{E}}_{{\rm{TF}}}\right)}_{\bullet ,n},{{\widetilde{O}}^{(i)}}_{\bullet ,n},{\theta }_{b}^{(i)}\right)$ is the predicted gene expression from the neural network of sample *n*. The neural network is parametrized by a set of weights and biases, collectively denoted by ${\theta }_{b}^{(i)}$. The weight *λ*_0_ is a tuning parameter.

The loss function of LINGER is composed of MSE, L1 regularization, manifold regularization and EWC loss: ${{\mathcal{L}}}_{{\rm{LINGER}}}={{{\lambda }}_{1}{\mathcal{L}}}_{{\rm{MSE}}}$ $+{{\lambda }}_{2}{{\mathcal{L}}}_{L1}+{{\lambda }}_{3}{{\mathcal{L}}}_{{\rm{Laplace}}}+{{{\lambda }}_{4}{\mathcal{L}}}_{{\rm{EWC}}}$. ${{\mathcal{L}}}_{{\rm{Laplace}}}$ represents the manifold regularization because a Laplacian matrix is used to generate this regularization term. The loss function terms correspond to gene *i*, and for simplicity, we omit subscripts $(i)$ for the chromatin accessibility matrix ($O$), parameters for the bulk model (${\theta }_{b}$) and parameters for LINGER (${\theta }_{l}$).

1. MSE
  $${{\mathcal{L}}}_{{\rm{MSE}}}\left({E}_{{\rm{TF}}},O,{E}_{i,\bullet },{\theta }_{l}\right)=\frac{1}{{N}_{{\rm{sc}}}}\mathop{\sum }\limits_{n=1}^{{N}_{{\rm{sc}}}}{\left(f\left({\left({E}_{{\rm{TF}}}\right)}_{\bullet ,n},{O}_{\bullet ,n},{\theta }_{l}\right)-{E}_{{in}}\right)}^{2}$$
  Here, ${E}_{{\rm{TF}}}\in {{\mathbb{R}}}^{{N}_{{\rm{TF}}}\times {N}_{{\rm{sc}}}}$ represents the TF expression matrix from the single-cell RNA-seq data, consisting of ${N}_{{\rm{sc}}}$ cells; $O\in {{\mathbb{R}}}^{{N}_{{\rm{RE}}}^{(i)}\times {N}_{{\rm{sc}}}}$ represents the RE chromatin accessibility matrix of the single-cell ATAC-seq data; $E\in {{\mathbb{R}}}^{{{N}_{{\rm{TG}}}\times N}_{{\rm{sc}}}}$ represents the expression of the genes across cells; and ${\theta }_{l}$ represents the parameters in the neural network. We use metacells to train the models; therefore, ${N}_{{\rm{sc}}}$ is the number of cells from metacell data.
2. L1 regularization
  $${ {\mathcal L} }_{L1}({E}_{\rm{TF}},O,{E}_{i,\cdot },{\theta }_{l})={\Vert {\theta }_{l}\Vert }_{1}$$
3. Laplacian loss (manifold regularization)
  We generate the adjacency matrix as: $${B}^{* }\in {{\mathbb{R}}}^{\left({N}_{{\rm{TF}}}+{N}_{{\rm{RE}}}^{(i)}\right)}$$$${\times \left({N}_{{\rm{TF}}}+{N}_{{\rm{RE}}}^{(i)}\right)}$$, where $${B}_{k,{N}_{{\rm{TF}}}+j}^{* }$$ and $${B}_{{N}_{{\rm{TF}}}+j,k}^{* }$$ represent the binding affinity of the TF $$k$$ and the RE $$j$$, which is elaborated in the following sections. $${L}^{{\rm{Norm}}}\in {{\mathbb{R}}}^{\left({N}_{{\rm{TF}}}+{N}_{{\rm{RE}}}^{(i)}\right)\times \left({N}_{{\rm{TF}}}+{N}_{{\rm{RE}}}^{(i)}\right)}$$ is the normalized Laplacian matrix based on the adjacency matrix.
  $${{\mathcal{L}}}_{{\rm{Laplace}}}\left({E}_{{\rm{TF}}},O,{E}_{i,\bullet },{\theta }_{l}\right)={tr}\left({\left({\theta }_{l}^{\left(1\right)}\right)}^{T}{L}^{{\rm{Norm}}}{\theta }_{l}^{\left(1\right)}\right)$$
  where ${\theta }_{l}^{\left(1\right)}\in {{\mathbb{R}}}^{\left({N}_{{\rm{TF}}}+{N}_{{\rm{RE}}}^{(i)}\right)\times 64}$ is the parameter matrix of the first hidden layer, which can capture the densely connected TF–RE modules.
4. EWC loss. EWC constrains the parameters of the first layer to stay in a region of ${\theta }_{b}^{\left(1\right)}$, which is previously learned from the bulk data^45^. To do so, EWC uses MSE between the parameters ${\theta }_{l}^{\left(1\right)}$ and ${\theta }_{b}^{\left(1\right)}$, weighted by the Fisher information, a metric of how important the parameter is, allowing the model to protect the performance, both for single-cell data and bulk data^45^.

$${{\mathcal{L}}}_{{\rm{EWC}}}\left({E}_{{\rm{TF}}},O,{E}_{i,\bullet },{\theta }_{l}\right)=\frac{1}{\left({N}_{{\rm{TF}}}+{N}_{{\rm{RE}}}\right)\times 64}\mathop{\sum }\limits_{i=1}^{{N}_{{\rm{TF}}}+{N}_{{\rm{RE}}}}\mathop{\sum }\limits_{j}^{64}{F}_{{ij}}{\left({\theta }_{l}^{\left(1\right)}\right)}_{i,\;j}{\left({\theta }_{b}^{\left(1\right)}\right)}_{i,\;j}$$

where $F$ is the fisher information matrix, which is detailed below, and ${\theta }_{l}^{\left(1\right)}\in {{\mathbb{R}}}^{\left({N}_{{\rm{TF}}}+K\right)\times 64}$ is the parameter matrix of the first hidden layer.

To construct a normalized Laplacian matrix, we first generate the TF–RE binding affinity matrix for all REs from the single-cell ATAC-seq data. We extract the REs 1 Mb from the TSS for the gene to be predicted. Let ${N}_{{\rm{RE}}}^{(i)}$ be the number of these REs and $B\in {{\mathbb{R}}}^{{N}_{{\rm{TF}}}\times {N}_{{\rm{RE}}}^{(i)}}$ be the TF–RE binding affinity matrix, where ${B}_{{kj}}$ represents the binding affinity for the TF $k$ and RE $j$. We construct a graph, taking TFs as the first ${N}_{{\rm{TF}}}$ nodes, REs as the remaining ${N}_{{\rm{RE}}}^{\;(i)}$ nodes and binding affinity as the edge weight between TF and RE. The edge weights of TF–TF and RE–RE are set to zero. Then the adjacency matrix ${B}^{* }\in {{\mathbb{R}}}^{\left({N}_{{\rm{TF}}}+{N}_{{\rm{RE}}}^{(i)}\right)\times \left({N}_{{\rm{TF}}}+{N}_{{\rm{RE}}}^{(i)}\right)}$ is defined as:

$$\begin{array}{l}{B}_{k,j}^{* }=\\\left\{\begin{array}{l}{B}_{k,j-{N}_{{\rm{TF}}}},k\in \left\{1,2,\ldots ,{N}_{{\rm{TF}}}\right\},j\in \{{N}_{{\rm{TF}}}+1,{N}_{{\rm{TF}}}+2,\ldots ,{N}_{{\rm{TF}}}+{N}_{{\rm{RE}}}^{(i)}\}\\ {B}_{j,k-{N}_{{\rm{TF}}}},k\in \left\{{N}_{{\rm{TF}}}+1,{N}_{{\rm{TF}}}+2,\ldots ,{N}_{{\rm{TF}}}+{N}_{{\rm{RE}}}^{\;(i)}\right\},:j\in \left\{1,2,\ldots ,{N}_{{\rm{TF}}}\right\}\\ 0,\qquad{\rm{else}}\end{array}\right.\end{array}$$

The Fisher information matrix is calculated based on the neural network trained on bulk data:

$$\begin{array}{l}{F}_{{ij}}={\rm{E}}\left[{\left(\displaystyle\frac{\partial }{\partial {\left({\theta }_{b}^{\left(1\right)}\right)}_{{ij}}}{{\mathcal{L}}}_{{\rm{MSE}}}\left({\widetilde{E}}_{{\rm{TF}}},\widetilde{O},{\widetilde{E}}_{i,\bullet },{\theta }_{b}\right)\right)}^{2}\right]\\\quad\;\;\;=\displaystyle\frac{1}{{N}_{b}}\mathop{\sum }\limits_{n=1}^{{N}_{b}}{\left(\displaystyle\frac{\partial}{\partial {\left({\theta }_{b}^{\left(1\right)}\right)}_{{ij}}}{\left(\frac{1}{{N}_{b}}f\left({\left({\widetilde{E}}_{{\rm{TF}}}\right)}_{\bullet ,n},{\widetilde{O}}_{\bullet ,n},{\theta }_{b}\right)-{\widetilde{E}}_{{in}}\right)}^{2}\right)}^{2}\end{array}$$

### GRN inference by Shapley value

The Shapley value measures the contribution of features in a machine-learning model and is widely used in algorithms such as deep learning, graphical models and reinforcement learning^85^. We use the average of absolute Shapley values across samples to infer the regulation strength of TF and RE to TGs, generating the RE–TG *cis*-regulatory strength and the TF–TG *trans*-regulatory strength. Let ${\beta }_{{ij}}$ represent the *cis*-regulatory strength of RE $j$ and TG *i*, and ${\gamma }_{{ki}}$ represent the *trans*-regulatory strength. To generate the TF–RE binding strength, we use the weights from the input layer (TFs and REs) to all nodes in the second layer of the neural network model to embed the TF or RE. The TF–RE binding strength is calculated by the PCC between the TF and RE based on this embedding. ${\alpha }_{{kj}}$ represents the TF–RE binding strength.

### Constructing cell type-specific GRNs

The TF–RE regulatory potential for a certain cell type is given by:

$${\rm{TFB}}_{{kj}}={C}_{{kj}}^{\;{s}_{k}}{{({E}_{{\rm{TF}}})}_{k}O}_{j}({\alpha }_{{kj}}+{B}_{{kj}})$$

where ${\rm{TFB}}_{{kj}}$ is the TF–RE regulation potential of TF $k$ and RE $j$; ${s}_{k}$ is an importance score of TF $k$ in the cell type to measure the preference of TF for activating cell type-specific open chromatin regions (which will be described in ‘TF importance score’ below); ${C}_{{kj}}$ is the PCC of TF $k$ and RE $j$; ${O}_{j}$ is the average chromatin accessibility across cells in the cell type; ${B}_{{kj}}$ is the binding affinity between TF $k$ and RE $j$; and ${\alpha }_{{kj}}$ is the TF–RE binding strength.

The RE–TG *cis*-regulatory potential is defined as:

$${\rm{CRP}}_{{ij}}={\beta }_{{ij}}{O}_{j}{E}_{i}{e}^{-\frac{{d}_{{ij}}}{{d}_{0}}}$$

where ${\rm{CRP}}_{{ij}}$ is the *cis*-regulatory potential of TG *i* and RE $j$; ${\beta }_{{ij}}$ is the *cis*-regulatory strength of RE $j$ and TG *i*; ${O}_{j}$ is the average chromatin accessibility; ${E}_{i}$ is the average gene expression across cells in the cell type; ${d}_{{ij}}$ is the distance between genomic locations of TG *i* and RE $j$; and ${d}_{0}$ is a fixed value used to scale the distance, which is set to 25,000 in this paper.

The TF–TG *trans*-regulatory potential is defined as the cumulative effect of corresponding REs on the TG:

$${\rm{TRP}}_{{ki}}={\gamma }_{{ki}}\sum _{j\in {S}_{i}}{\rm{TFB}}_{{kj}}{\rm{CRP}}_{{ij}}$$

where ${\gamma }_{{ki}}$ is the TF–TG *trans*-regulatory strength of TF $k$ and TG *i*; ${S}_{i}$ is the set of REs within 1 Mb from the TSS for TG *i*; ${\rm{CRP}}_{{ij}}$ is the *cis*-regulatory potential of TG *i* and RE $j$; and ${\rm{TFB}}_{{kj}}$ is the TF–RE regulation potential of TF $k$ and RE $j$.

### Constructing cell-level GRNs

Cell-level GRNs are inferred by integrating information consistent across all cells, such as regulatory strength, binding affinity and RE–TG distance, with cell-level information, such as gene expression and chromatin accessibility. This approach is similar to inferring cell type-specific GRNs, with the key difference that cell-level GRNs use cell-level TF expression ${E}_{{\rm{TF}}}$, chromatin accessibility $O$ and gene expression $E$ rather than cell type-averaged data. This allows us to infer the network for each individual cell based on its specific characteristics rather than grouping cells into predefined types.

### TF importance score

To systematically identify TFs playing a pivotal role in controlling the chromatin accessibility of cell type, we introduce a TF importance score. The score is designed to measure the preference of TFs for activating cell type-specific REs. The input is multiome single-cell data with known cell type annotations. There are four steps to generate the TF importance score:

1. Motif enrichment. We perform the motif enrichment analysis^86^ to identify the motifs significantly enriched in the binding sites of the top 5,000 cell type-specific REs. We use the *P* value to measure the significant level of motif enrichment.
2. TF–RE correlation. To avoid dropouts in single-cell data, we recover the original count matrix by an average of the observed count of nearby cells. We calculate PCC between the TF expression and cell type-specific RE chromatin accessibility, with ${r}_{{kj}}$ representing the PCC of the TF $k$ and the RE $j$. To mitigate the bias in the distribution of TF expression and REs chromatin accessibility so that the PCC is comparable across different TF–RE pairs, we permute the cell barcode in the gene expression data and then calculate, generating a background PCC distribution for each TF–RE pair. We generate a *z*-score for ${r}_{{kj}}$,
  $${z}_{{kj}}=\frac{{r}_{{kj}}-{\mu }_{{kj}}}{{\sigma }_{{kj}}}$$
  where ${\mu }_{{kj}}$ and ${\sigma }_{{kj}}^{2}$ are the mean and the variance of the background PCC distribution between ${\rm{TF}}_{k}$ and ${\rm{RE}}_{j}$.
3. The co-activity score of the TF-motif pair. To pair TFs with their motifs, we match 713 TFs and 1,331 motifs, yielding 8,793 TF-motif pairs^84^. Let $\left(k,m\right)$ denote the TF-motif pair of TF $k$ and motif $m$. We then calculate a co-activity score for a TF-motif pair for $\left(k,m\right)$, defined as the average *z*-score across cell type-specific REs with at least one motif binding site. That is ${z}_{k,m}^{\;{co}}=\frac{1}{{N}_{m}}\sum _{j\in {\left\{{\rm{RE}}\;\right\}}_{m}}{z}_{{kj}}$, where ${\left\{{\rm{RE}}\right\}}_{m}$ is the set of REs with the $m$-th motif binding; and ${N}_{m}=\left|{\left\{{\rm{RE}}\right\}}_{m}\right|$ is the number of REs in ${\left\{{\rm{RE}}\right\}}_{m}$.
4. TF importance score. The score of the TF-motif pair, $\left(k,m\right)$, is given by:

$${s}_{(k,m)}=\left\{\begin{array}{cc}{z}_{(k,m)}^{\;{co}}, & {\rm{if}}\,{p}_{m} < 0.05\\ {\rm{NA}}, & {\rm{otherwise}}\end{array}\right.$$

where ${p}_{m}$ is the *P* value of the $m$^th^ motif from the motif-enrichment analysis and ${s}_{(k,m)}$ is the importance score of the TF-motif pair $(k,m)$. The TF importance score for the TF $k$ is the average TF-motif pair TF importance score across motifs, omitting NA:

$${s}_{k}=\left\{\begin{array}{cc}\frac{1}{{N}_{\left(k,m\right)}}\sum _{m\in \left\{{{m|s}}_{\left(k,m\right)}\ne {\rm{NA}}\right\}}{s}_{\left(k,m\right)}, & {\rm{if}}\,{N}_{\left(k,m\right)} > 0\\ 0, & {\rm{if}}\,{N}_{\left(k,m\right)}=0\end{array}\right.,$$

where ${N}_{(k,m)}=\left|\{{{m|s}}_{(k,m)}\ne {\rm{NA}}\}\right|$ is the number of the TF-motif pair of the TF $k$, whose CECI score is not NA.

### TF–RE binding affinity matrix

We download 713 TF position weight matrices for the known motifs from GitHub page of PECA2^84^, which is collected from widely used databases including JASPAR, TRANSFAC, UniPROBE and Taipale. Given a list of REs, we calculate the binding affinity score for each TF by motif scan using Homer^86^, as a quantitative measure of the strength of the interaction between TF and RE^20^.

### Identify motif-binding REs

We identify the REs with motif binding by motif scan using Homer^86^.

### ChIP–seq-based validation

Given that the choice of TFs for benchmarking may affect the final results, we use the following standard to collect all ChIP–seq data from the Cistrome database that satisfies the following criteria.

The procedure for choosing ChIP–seq data for PBMC is as follows.

- We downloaded all human TF ChIP–seq information, including 11,349 datasets.
- We filtered samples that did not pass quality control, and 4,657 datasets remained.
- We chose samples in blood tissue, and 609 datasets remained.
- We filtered the cell line data that is not consistent with PBMC cell types, and 63 datasets remained.
- We chose the TF expressed in single-cell data and with known motifs available, and 39 datasets remained.
- We chose the experiments that were done in one of the 14 cell types detected in the PBMC data, and 20 datasets remained.

The procedure for choosing ChIP–seq data for the H1 cell line is as follows:

- We downloaded all human TF ChIP–seq information, including 11,349 datasets.
- We filtered samples that did not pass quality control, and 4,657 datasets remained.
- We chose the H1 cell line, and 42 datasets remained.
- We chose the TF expressed in single-cell data and with known motifs available, and 33 datasets remained.

### Perturbation-based validation

The criteria for choosing ground truth from the KnockTF database is similar to ChIP–seq data.

The procedure for choosing knockdown data for PBMC is as follows.

- We selected the molecular type as ‘TF’ and chose the ‘Peripheral_blood’ tissue type, with 21 cases remaining.
- There are 11 datasets included in the PBMCs cell type in the single-cell data.
- We chose the TF expressed in single-cell data and with known motifs available, and 8 datasets remained.

### PBMC 10× data

We download the PBMC 10K data from the 10× Genomics website (https://support.10xgenomics.com/single-cell-multiome-atac-gex/datasets). Note that it contains 11,909 cells, and the granulocytes were removed by cell sorting of this dataset. We use the filtered cells by features matrix from the output of 10× Genomics software Cell Ranger ARC as input and perform the downstream analysis. First, we perform weighted nearest neighbor analysis in Seurat (version 4.0)^87^, and it removes 1,497 cells. We also remove the cells that do not have surrogate ground truth and it results in 9,543 cells. We generate metacells data by randomly selecting the square root of the number of cells in each cell type and averaging the expression levels and chromatin accessibility of the 100 nearest cells to produce the gene expression and chromatin accessibility values of the selected cells. The metacells data were directly input into LINGER for analysis.

### AUPR ratio

To measure the accuracy of a predictor, we defined the AUPR ratio as the ratio of the AUPR of a method to that of a random predictor. For a random predictor, the AUPR equals the fraction of positive samples in the dataset. The AUPR ratio is defined as ${\rm{AUPR}\frac{\#\,{sample}}{\#\,{real}\,{positive}}}$, representing the fold change of the accuracy of a predictor compared to the random prediction.

### LINGER reveals the regulatory landscape of GWAS traits

We propose a method to integrate GWAS summary statistics data and cell type-specific GRNs to identify the relevant cell types, key TFs and sub-GRNs responsible for GWAS variants. To identify relevant cell types, we first project the risk SNP identified from GWAS summary data to a gene. We then link the gene within the 200 kb region centering on the SNP and assign the most significant *P* value of linked SNPs to each gene. In this study, the trait-related genes are defined as those with *P* < 0.01 after multiple testing adjustments. We then calculate a trait regulation score for each TF in each cell type, measuring the enrichment of GWAS genes downstream of the TF based on the cell type-specific GRN. We choose 1,000 top-ranked genes according to the *trans*-regulation as the TG of each TF and count the number of overlapping genes with trait-related genes. The enrichment of cell types to the GWAS traits is measured by a *t*-test comparing the number of overlapping genes between the 100 top-expressed and 100 randomly chosen TFs.

To identify key TFs of GWAS traits, we combine the trait regulation score and the gene expression level of TFs in each cell type. The trait regulation score is the *z*-score of the number of overlapping genes of a TF across all TFs. The expression level is also transformed to a *z*-score based on the gene expression. The final importance of key TFs is the summation of the expression level and trait regulation score.

### Identify driver regulators based on transcription profiles

To measure the activity of each TF on the independent transcriptional profiles, we first constructed a TG set for each TF based on the corresponding GRN. We perform quantile normalization to the *trans*-regulation score of each gene across all TFs. We then rank the genes for each TF and choose the top 1,000 genes as the target. Next, we use the R package AUCell^22^ to calculate whether the TGs are enriched within the expressed genes for each sample, which defines the TF activity.

### Benchmark the *trans*-regulatory potential

We compare LINGER’s performance of the *trans*-regulation prediction using PCC, SCENIC+, GENIE3 and PIDC as competitors to LINGER. Owing to the time-consuming nature of PIDC’s mutual information-based algorithm, we used the 5,000 most variable genes as input. As a result, there are 9 TFs and 14 TFs in ground truth data left for PBMCs and the H1 cell line, respectively.

### Reporting summary

Further information on research design is available in the Nature Portfolio Reporting Summary linked to this article.

## Online content

Any methods, additional references, Nature Portfolio reporting summaries, source data, extended data, supplementary information, acknowledgements, peer review information; details of author contributions and competing interests; and statements of data and code availability are available at 10.1038/s41587-024-02182-7.

## Supplementary information

Reporting Summary

Supplementary Tables 1–8 Table 1: Information of ground truth data for the *trans*-regulation and TF–RE binding potential for PBMC data. Table 2: Details of eQTL data as ground truth for the *cis*-regulation for PBMCs. Table 3: Functional enrichment of *cis*-regulatory dominant gene. Table 4: Ground truth data for the *trans-*regulation and TF-RE binding potential for H1 cell line. Table 5: Details of Hi-C data as ground truth for the *cis*-regulation for PBMCs. Table 6: Details of Fig. 3b,c Table 7: Details of Fig. 4c,d. Table 8: Ground truth data information of the *trans*-regulation for PBMC data from the KnockTF database.

## Data Availability

The PBMC data used during this study was downloaded from the 10× Genomics website (https://s3-us-west-2.amazonaws.com/10x.files/samples/cell-arc/1.0.0/pbmc_granulocyte_sorted_10k/pbmc_granulocyte_sorted_10k_fastqs.tar)^40^. SNARE-seq was downloaded from NCBI Gene Expression Omnibus (https://www.ncbi.nlm.nih.gov/geo/) under accession number GSE126074 (ref. ^55^).

## Code availability

The software is available at GitHub^88^ (https://github.com/Durenlab/LINGER) and the Zenodo repository under the GPLv3 license^89^. We used Python and R for this study.
