# Supplementary material for: Inferring gene regulatory networks from single-cell multiome data using atlas-scale external data
Source: Nat Biotechnol. 2024 Apr 12;43(2):247–57. doi: 10.1038/s41587-024-02182-7 (PMC11825371; doi:10.1038/s41587-024-02182-7)
Supplement: Supplementary file 1 — Reporting Summary [file 41587_2024_2182_MOESM1_ESM.pdf]

nature portfolio

Corresponding author(s): Zhana Duren

Last updated by author(s): Feb 13, 2024

Reporting Summary

Nature Portfolio wishes to improve the reproducibility of the work that we publish. This form provides structure for consistency and transparency in reporting. For further information on Nature Portfolio policies, see our Editorial Policies and the Editorial Policy Checklist.

Statistics

For all statistical analyses, confirm that the following items are present in the figure legend, table legend, main text, or Methods section.

n/a	Confirmed	
☐	☒	The exact sample size (*n*) for each experimental group/condition, given as a discrete number and unit of measurement
☐	☒	A statement on whether measurements were taken from distinct samples or whether the same sample was measured repeatedly
☐	☒	The statistical test(s) used AND whether they are one- or two-sided<br>*Only common tests should be described solely by name; describe more complex techniques in the Methods section.*
☒	☐	A description of all covariates tested
☐	☒	A description of any assumptions or corrections, such as tests of normality and adjustment for multiple comparisons
☐	☒	A full description of the statistical parameters including central tendency (e.g. means) or other basic estimates (e.g. regression coefficient) AND variation (e.g. standard deviation) or associated estimates of uncertainty (e.g. confidence intervals)
☐	☒	For null hypothesis testing, the test statistic (e.g. *F*, *t*, *r*) with confidence intervals, effect sizes, degrees of freedom and *P* value noted<br>*Give P values as exact values whenever suitable.*
☒	☐	For Bayesian analysis, information on the choice of priors and Markov chain Monte Carlo settings
☒	☐	For hierarchical and complex designs, identification of the appropriate level for tests and full reporting of outcomes
☐	☒	Estimates of effect sizes (e.g. Cohen's *d*, Pearson's *r*), indicating how they were calculated

Our web collection on statistics for biologists contains articles on many of the points above.

Software and code

Policy information about availability of computer code

Data collection

No code was used to collect data for this study.

Data analysis

The software, package, and library used in this study are listed as follows:
We use the following Python packages with python 3.10.9:
torch: 1.13.1+cu117; numpy: 1.23.5; scipy: 1.10.1; pandas: 1.5.3; sklearn: 1.2.1; shap: 0.41.0; joblib: 1.2.0; scenicplus: 0.1.dev466+g37cf1fc.
We use conda: 4.12.0; bedtools: 2.26.0, homer: v4.11.1.
We use the following R packages with R 4.2.2:
AUCell: 1.20.2; biomaRt: 2.54.1; cgdsr: 1.3.0; cowplot: 1.1.1; egg: 0.4.5; ggplot2: 3.4.2; ggpubr: 0.6.0; limma: 3.54.2; Matrix: 1.5-4; survival: 3.5-5; pROC: 1.18.0; PRROC: 1.3.1; grid: 4.2.2; tidyr: 1.3.0; MASS: 7.3-59; viridis: 0.6.2; RColorBrewer: 1.1-3, GENIE3: 1.20.0.

The software is available at GitHub: https://github.com/Durenlab/LINGER

For manuscripts utilizing custom algorithms or software that are central to the research but not yet described in published literature, software must be made available to editors and reviewers. We strongly encourage code deposition in a community repository (e.g. GitHub). See the Nature Portfolio guidelines for submitting code & software for further information.

Data

Policy information about availability of data

All manuscripts must include a data availability statement. This statement should provide the following information, where applicable:

- Accession codes, unique identifiers, or web links for publicly available datasets
- A description of any restrictions on data availability
- For clinical datasets or third party data, please ensure that the statement adheres to our policy

The PBMCs data used during this study is downloaded from the 10X Genomics website (https://s3-us-west-2.amazonaws.com/10x.files/samples/cell-arc/1.0.0/pbmc_granulocyte_sorted_10k/pbmc_granulocyte_sorted_10k_fastqs.tar). SNARE-seq is downloaded from NCBI Gene Expression Omnibus (GEO, https://www.ncbi.nlm.nih.gov/geo/) under accession number GSE126074.

Human research participants

Policy information about studies involving human research participants and Sex and Gender in Research.

Reporting on sex and gender	Not relevant to our study, since we do not attempt to draw biological conclusions pertaining to sex or gender. We apply the method to expression data from male and female donors. There are no sex- or gender-based analyses in this manuscript.
Population characteristics	Not relevant to our study. We use published single cell data and bulk data from ENCODE Project, in which this information is unknown. In addition, we collect the independent gene expression data by collect all samples released from the publisher studies.
Recruitment	This study did not involve recruitment of samples.
Ethics oversight	Not relevant to our study.

Note that full information on the approval of the study protocol must also be provided in the manuscript.

Field-specific reporting

Please select the one below that is the best fit for your research. If you are not sure, read the appropriate sections before making your selection.

☒ Life sciences ☐ Behavioural & social sciences ☐ Ecological, evolutionary & environmental sciences

For a reference copy of the document with all sections, see nature.com/documents/nr-reporting-summary-flat.pdf

Life sciences study design

All studies must disclose on these points even when the disclosure is negative.

Sample size	No sample size calculation is performed. The sample size depends on the availability of datasets.
Data exclusions	We filter the original PBMC data to remove the cells with poor quality. First, we perform Seurat 4.0 weighted nearest neighbor (WNN) analysis, and it removes 1497 cells. We also remove the cells that don't have surrogate ground truth, and it results in 9543 cells.
Replication	We validate our method to 2 datasets and compare with other methods. All attempts were successful.
Randomization	No relevant to our study, since our method assumes the group annotation of samples is known.
Blinding	The data collection and analyses are performed by same investigator. The group information and data are collected together. Therefore, analyses are not blinded.

Reporting for specific materials, systems and methods

We require information from authors about some types of materials, experimental systems and methods used in many studies. Here, indicate whether each material, system or method listed is relevant to your study. If you are not sure if a list item applies to your research, read the appropriate section before selecting a response.

Materials & experimental systems

n/a	Involved in the study
☒	☐ Antibodies
☒	☐ Eukaryotic cell lines
☒	☐ Palaeontology and archaeology
☒	☐ Animals and other organisms
☒	☐ Clinical data
☒	☐ Dual use research of concern

Methods

n/a	Involved in the study
☒	☐ ChIP-seq
☒	☐ Flow cytometry
☒	☐ MRI-based neuroimaging
